# Brief dataset on chemical and mechanical properties of Corypha utan leaf fiber-reinforced composite with alkaline and silane treatment

**DOI:** 10.1016/j.dib.2021.107714

**Published:** 2021-12-13

**Authors:** Kholqillah Ardhian Ilman, Agung Setyo Darmawan, Muhammad Ali Rofiq, Yan Anton Prayoga, Iman Nasucha, Rudy Faizal

**Affiliations:** a Department of Mechanical Engineering, Faculty of Engineering, Universitas Muhammadiyah Surakarta, Indonesia; b Department of Civil Engineering, Faculty of Engineering, Universitas Muhammadiyah Surakarta, Indonesia

**Keywords:** Composite, Chemical properties, Mechanical properties, Natural fiber, FTIR

## Abstract

This paper presents the chemical and mechanical properties of Corypha utan leaf fiber (CULF) subjected to different chemical treatments for reinforced composite materials. Natural fibers are currently considered as an alternative constituent for composite reinforcement due to their friendly environment character. The CULF were chemically treated during a pre-fabrication process using NaOH, and 3-(Trimethoxysilyl) propyl methacrylate was introduced at three different concentrations of 0%, 5%, and 10%, respectively. All the chemical treatments on CULF were observed using Fourier Transform Infrared (FTIR) Spectroscopy. Furthermore, vacuum bagging method with unsaturated polyester was used to manufacture CULF as composite reinforcement. Tensile and bending tests were carried out to collect mechanical properties data of the produced CULF reinforced composites. The data obtained could support further study in the area and development of natural fiber-reinforced composite.

## Specification Table

**Table:** 

Subject	Composite, Engineering, Natural Fiber
Specific subject area	Chemical properties of natural fiber and mechanics of material.
Type of data	Plain text files
How data were acquired	Instruments: Fourier Transform Infrared Spectroscopy (FTIR), Tensilone RTF-2410 Universal Testing Machine
Data format	Raw
Parameters for data collection	Corypha utan leaf fibers were subjected to alkali treatment at two different concentrations of 0% and 4% (w/w). Silane treatments were introduced on the fibers at three concentration percentages (0, 5, and 10 wt. %). The fibers were manufactured as reinforced composites with unsaturated polyester as the matrix. The fiber-to-matrix weight ratio was kept at an average of 26.5%.
Description for data collection	Information of used wavenumber and percentage of absorbed transmittance on untreated and chemically treated Corypha utan leaf fibers were collected using Fourier Transform Infrared Spectroscopy (FTIR). The obtained variables are wavenumbers and transmittance. Tensile and bending test results were acquired using Universal Testing Machine from zero loading until the specimens were fractured. Obtained variables from this test are crosshead displacement and applied load.
Data source location	Universitas Muhammadiyah Surakarta, Indonesia
Data accessibility	Repository name: Mendeley Data Data identification number: DOI: 10.17632/jwmkg444gf.2 Direct URL to Data: https://doi.org/10.17632/jwmkg444gf.2
Related research article	K.A. Ilman, J. Jamasri, K. Kusmono, H. Hestiawan, Effect of stacking sequences and silane treatments on the mechanical properties of agel leaf/jute/glass fiber-reinforced hybrid composite, Compos. Mech. Comput. Appl. An Int. J. 9 (2018) 311-329. https://doi.org/10.1615/compmechcomputapplintj.2018025443[1]

## Value of the Data

• The raw data is useful for further study related to chemical treatment on natural fibers and its application on composites material, especially its effect on mechanical properties of composite with hydrophobic matrix.
• People involved in the evaluation, development, and production of natural fiber-reinforced composite products and components will find beneficial information from the raw data.
• The data can be re-used to compare various effects of alkali and silane treatment concentrations on natural fibers, especially with the hydrophobic and hydrophilic properties.

## 1 Data Description

This paper comprises two datasets categories: Fourier Transform Infrared Spectroscopy (FTIR) and the mechanical test results. The four results obtained from FTIR for the untreated and treated CULF composites are described in two columns: wavenumber (in cm^−1^) and transmittance (in %) for each file. The three Results of the tensile and bending test are described in 3 columns: specimen number (sample identifier), crosshead displacement (in mm), and applied load (in N) for each file. Tensile and bending tests were executed using Tensilone RTF-2410 Universal Testing Machine. The files of all results obtained are described in Table 1 as follows.

**Table 1:** Description for each given file.

File Name	Description
F.0AL-0SIL.txt	FTIR data of pure (untreated) CULF
F.4AL-0SIL.txt	FTIR data of CULF with 4% (w/w) alkali treatment
F.4AL-5SIL.txt	FTIR data of CULF with 4% (w/w) alkali and 5wt.% silane treatment
F.4AL-10SIL.txt	FTIR data of CULF with 4% (w/w) alkali and 10wt.% silane treatment
0.A_T_1.xls	Tension-test data for pure CULF-reinforced composite
5.A_T_1.xls	Tension-test data for 4% alkaline+5% silane treated CULF-reinforced composite
10.A_T_1.xls	Tension-test data for 4% alkaline+10% silane treated CULF-reinforced composite
0.A_B_1.xls	3-point bending test data for pure CULF-reinforced composite
5.A_B_1.xls	3-point bending test data for 4% alkaline+5% silane treated CULF-reinforced composite
10.A_B_1.xls	3-point bending test data for 4% alkaline+10% silane treated CULF-reinforced composite

## 2 Experimental Design, Materials and Methods

The composite specimens were composed of two constituents (reinforcement and matrix). While the reinforcement was made of natural fiber derived from a plant called Corypha utan in woven form, unsaturated polyester (YUKALAC 157 BQTN-EX) was chosen as the matrix. The vacuum bagging method was used to fabricate the specimens with a fiber-to-matrix weight ratio kept at an average of 26.5%. Tensile and bending test specimens were fabricated following ASTM D638-03 specimen type 1 [2] and ASTM D790-03 [3]. Both tensile and bending test results were acquired using Universal Testing Machine Tensilone RTF-2410 from zero loading conditions until the specimens were fractured.

Various chemical treatment (alkali-silane) was introduced as a pre-fabrication process. Corypha utan fibers were alkali-treated using 4% (w/w) NaOH, and silane of 3-(Trimethoxysilyl) propyl methacrylate was used with three different concentrations (0, 5, and 10 wt. %.) on the surface of the fibers. The wavenumber and percentage of every chemically treated fiber's absorbed transmittance were acquired using Fourier Transform Infrared Spectroscopy (FTIR).

## CRediT authorship contribution statement

**Kholqillah Ardhian Ilman:** Conceptualization, Methodology, Writing – original draft. **Agung Setyo Darmawan:** Supervision. **Muhammad Ali Rofiq:** Validation, Writing – review & editing. **Yan Anton Prayoga:** Data curation. **Iman Nasucha:** Resources. **Rudy Faizal:** Investigation.

## Declaration of Competing Interest

The authors certify that they have no conflict in financial interests or personal relations which have impacted the work described in this paper.

## Appendix Supplementary materials

Image, application 1

Image, application 2

Image, application 3

Image, application 4

Image, application 5

Image, application 6

Image, application 7

Image, application 8

Image, application 9

Image, application 10
